# In Silico Characterization and Structural Modeling of* Dermacentor andersoni* p36 Immunosuppressive Protein

**DOI:** 10.1155/2018/7963401

**Published:** 2018-04-08

**Authors:** Martin Omulindi Oyugi, Johnson Kangethe Kinyua, Esther Nkirote Magiri, Milcah Wagio Kigoni, Evenilton Pessoa Costa, Naftaly Wang'ombe Githaka

**Affiliations:** ^1^Department of Biochemistry, Jomo Kenyatta University of Agriculture and Technology, P.O. Box 62000, Nairobi 00200, Kenya; ^2^Cooperative University of Kenya, P.O. Box 24814, Nairobi 00502, Kenya; ^3^Department of Biochemistry, Kenyatta University, P.O. Box 43844, Nairobi 00100, Kenya; ^4^Unit of Animal Experimentation, State University of North Fluminense, Centre of Biosciences and Biotechnology, Campos dos Goytacazes, RJ, Brazil; ^5^Animal and Human Health Program, International Livestock Research Institute, P.O. Box 30709, Nairobi 00100, Kenya

## Abstract

Ticks cause approximately $17–19 billion economic losses to the livestock industry globally. Development of recombinant antitick vaccine is greatly hindered by insufficient knowledge and understanding of proteins expressed by ticks. Ticks secrete immunosuppressant proteins that modulate the host's immune system during blood feeding; these molecules could be a target for antivector vaccine development. Recombinant p36, a 36 kDa immunosuppressor from the saliva of female* Dermacentor andersoni*, suppresses T-lymphocytes proliferation* in vitro. *To identify potential unique structural and dynamic properties responsible for the immunosuppressive function of p36 proteins, this study utilized bioinformatic tool to characterize and model structure of* D. andersoni* p36 protein. Evaluation of p36 protein family as suitable vaccine antigens predicted a p36 homolog in* Rhipicephalus appendiculatus*, the tick vector of East Coast fever, with an antigenicity score of 0.7701 that compares well with that of Bm86 (0.7681), the protein antigen that constitute commercial tick vaccine Tickgard™. Ab initio modeling of the* D. andersoni* p36 protein yielded a 3D structure that predicted conserved antigenic region, which has potential of binding immunomodulating ligands including glycerol and lactose, found located within exposed loop, suggesting a likely role in immunosuppressive function of tick p36 proteins. Laboratory confirmation of these preliminary results is necessary in future studies.

## 1. Introduction

Ticks are considered among the most important vectors of livestock diseases worldwide as well as major vectors of pet diseases [1]. In tropical Africa, ticks and the tick-transmissible diseases constitute a major obstacle to livestock development [2]. Like elsewhere in the world, chemical acaricides have been the mainstay of tick control in this region; however, increasing resistance to this group of insecticides threatens livestock production systems, especially small-holding sectors that rely on rearing of exotic cattle breeds that are more susceptible to tick infestation and tick-borne diseases [TBDs] [3]. Integrated tick control incorporating reduced acaricide use, breeding cattle for tick resistance, rotational grazing, and use of vaccines presents a sustainable and long-term strategy to the control of ticks and TBDs in the tropics [4].

Numerous studies have shown the potential of immunological methods to control tick infestation by targeting critical tick physiological processes. Existing antitick vaccines work by eliciting humoral and cellular responses against tick cell membrane antigens [5, 6]. Vaccines capable of quelling both the arthropod vector and disease-causing pathogens are also under development [7]. Despite clear advantages of controlling ticks through vaccination, this strategy is presently hampered by antigenic sequence variations between geographically isolated tick populations and species causing vaccine resistance in some regions [8] and lack of efficacy in others [9]. These limitations necessitate search of alternative antigens for inclusion in the next-generation tick vaccines.

Proteins found in tick saliva play critical roles during blood meal acquisition [10]. The pharmacologically active components secreted in their saliva help ticks circumvent host defenses such as haemostatic and immune responses of the host, thereby enabling blood feeding in hematophagous arthropods [11]. One such class of biological compounds is immunosuppressant proteins, which modulate the host's immune system during tick's blood feeding [12], making them suitable target in the search of novel vaccines against arthropod-transmitted diseases [13]. Low molecular weight proteins 5–36 kDa from tick saliva proteins have been shown to inhibit T-lymphocytes proliferation* in vitro *[14]. Active immunization of mice with Salp15, a 15 kDa secreted salivary gland protein from* I. scapularis*, showed substantial protection (60%) from tick-borne* Borrelia *[15]. Tick subolesin (SUB), the ortholog of insect and vertebrate akirins (AKR), was discovered as a tick protective antigen in* Ixodes scapularis *[16]. Vaccines containing conserved SUB/AKR protective epitopes have been shown to protect against tick, mosquito, and sandfly infestations, thus suggesting the possibility of developing universal vaccines for the control of arthropod vector infestations [17].

Protein antigens conserved across vector species could be used in developing cross-protective vaccines against multiple arthropod vectors and their associated pathogens [17, 18]. Alarcon-Chaidez et al. [19] cloned and characterized a 36 kDa immunosuppressive protein p36 from the salivary glands of partially engorged, female* D. andersoni*, that suppressed Con-A induced* in vitro* proliferation of normal murine T-lymphocytes by more than 90% [20]. Genes related to* D. andersoni*-derived p36 gene, such as Ra-p36, Av-p36, Hl-p36, and Rhp36, have been reported in* A. variegatum *[21],* R. appendiculatus *[22],* H. longicornis *[23], and* R. haemaphysaloides* [24]. Most proteins are, however, not sufficiently protective on their own suggesting the need for a multiantigen/chimeric vaccine that incorporates critical tick and pathogen antigenic epitopes [16, 25] to elicit synergistic antipathogen and antitick immune responses. Computational characterization and 3D structure modeling of* D. andersoni* p36 protein undertaken by this study is an initial step in understanding molecular basis of immune recognition which is a challenge in vaccine development [26]. The p36 conserved antigenic region predicted by this study has binding residues for ligands like glycerol and lactose which are associated with an immunomodulatory role suggesting this site may have a role in suppression of select T-cell receptor induced signaling events of* D. andersoni* p36 and its related proteins.

## 2. Methods

### 2.1. Sequence Characterization of Tick p36 Proteins

All tick proteins deposited in National Centre for Biotechnology Information (NCBI) (https://www.ncbi.nlm.nih.gov) protein database were retrieved and deposited in a standalone MySQL based database (https://www.mysql.com).* D. andersoni* p36 protein was used as reference sequence in conducting homology searches, Blastp [27] and OrthoMCL [28, 29], of tick proteins in the database.

The identified tick p36-related proteins were subjected to MEME tool search (http://meme-suite.org/tools/meme) to predict conserved motifs characteristic of p36 proteins. Motif search tool (http://www.genome.jp/tools/motif) then searched for function of identified common motifs in the database of known motifs. Tick p36 protein sequences were then aligned by a multiple sequence alignment tool, Clustal Omega (https://www.ebi.ac.uk/Tools/msa/clustalo). Phylogenetic tree construction was by maximum likelihood method [30] and evolutionary distance computed using Poisson correction method [31]. Bootstrap resampling (1000 replicates) assessed robustness of the groupings.

### 2.2. Identification of Antigenic Determinants in the p36 Proteins

SignalP 4.1 (http://www.cbs.dtu.dk/services/SignalP), TMHMM (http://www.cbs.dtu.dk/services/TMHMM), and PredGPI (http://gpcr.biocomp.unibo.it/predgpi) servers were used to determine if tick p36 proteins are preferably secretory, transmembrane, or have a glycosylphosphatidylinositol (GPI) sites, respectively. Antigenic potentials of tick p36 proteins against reference Bm86, a known antitick vaccine antigen, were evaluated by vaxijen tool (http://www.ddg-pharmfac.net/vaxijen/VaxiJen/VaxiJen.html); the model selected was parasite whose standard threshold is 0.5000. The antigenic regions of* D. andersoni* p36 and other p36 proteins predicted with an antigenic score above 0.7000 were mapped by online tools that predict antigenic peptides (Immunomedicine) (http://imed.med.ucm.es/Tools/antigenic.pl) and SVMTrip [32]. Immunogenic segments/residues of the predicted antigenic region were identified by an online Epitopia tool (http://epitopia.tau.ac.il). Sprint-Pep tool (http://sparks-lab.org/server/SPRINT) was then used to predict protein-peptide binding sites while Coach tool (https://zhanglab.ccmb.med.umich.edu/COACH) predicted ligands likely to bind these sites found within the region predicted as a potential p36 protein conserved site.

### 2.3. Structural Modeling of* D. andersoni* p36 Protein

Physicochemical properties of* D. andersoni *p36 protein were analyzed by ExPASyProtParam (https://www.expasy.org) server while its secondary structure was characterized by online tool Spider^2^ (http://sparks-lab.org/yueyang/server/SPIDER2). The crystal or NMR structure of tick p36 protein is currently not available in the protein data bank (PDB) (https://www.rcsb.org/pdb/). The 3D structure of* D. andersoni* p36 protein was developed by QUARK ab initio modeling [33] that builds 3D structure from “Scratch,” based on physical principles rather than previously solved structures. 10 models, designated as 1, 2, 3, 4, 5, 6, 7, 8, 9, and 10, were generated and validated by analyzing Verify 3D scores [34] of Ramachandran plots for each model. Based on the scores, models 2 and 9 were selected as likely 3D structures of* D. andersoni *p36 protein because they scored 81.41% and 88.44%, respectively, meeting Verify 3D validation tool limit of 80% of the amino acids residues scoring >=0.2 in the 3D/1D profile.

The two selected models had their atomic structures refined by ModRefiner [35] after which their respective generated Ramachandran plots were validated by RAMPAGE (http://mordred.bioc.cam.ac.uk/~rapper/rampage.php) and ProQ (https://proq.bioinfo.se/ProQ/ProQ.html). The validation scores guided selection of model 2 as the best 3D structure of* D. andersoni *p36 protein. PDBsum (https://www.ebi.ac.uk/thornton-srv/databases/cgi-bin/pdbsum/GetPage.pl?pdbcode=index.html) was used to check location of predicted conserved antigenic region in the 3D structure of* D. andersoni* p36 protein.

## 3. Results and Discussion

### 3.1. Identification and Phylogenetic Analysis of p36 Proteins from Ixodid Ticks

The study identified 32 homologs of* D. andersoni* p36 protein among 6 ixodid (hard) tick species (Table 1). These included p36 genes reported in earlier studies from* R. appendiculatus*,* A. variegatum*,* H. longicornis*, and* Rhipicephalus haemaphysaloides *tick species as well as those found by this study in* Amblyomma sculptum* and* Amblyomma aureolatum*. Among these homologs, 4 co-orthologs which are potential orthologs of* D. andersoni* p36 protein were identified in* R. appendiculatus* species (Table 1). Occurrence of p36 protein across a range of tick species may be related to a biological function for this protein in tick feeding [36]. Several p36 immunosuppressant protein sequences were found in a single tick species suggesting functional and structural redundancy in which a tick expresses multiple similar proteins in minute quantities during feeding [37]. Such redundancy may render saliva proteins less immunogenic, as reported with cystatins [38].

**Figure 1:**
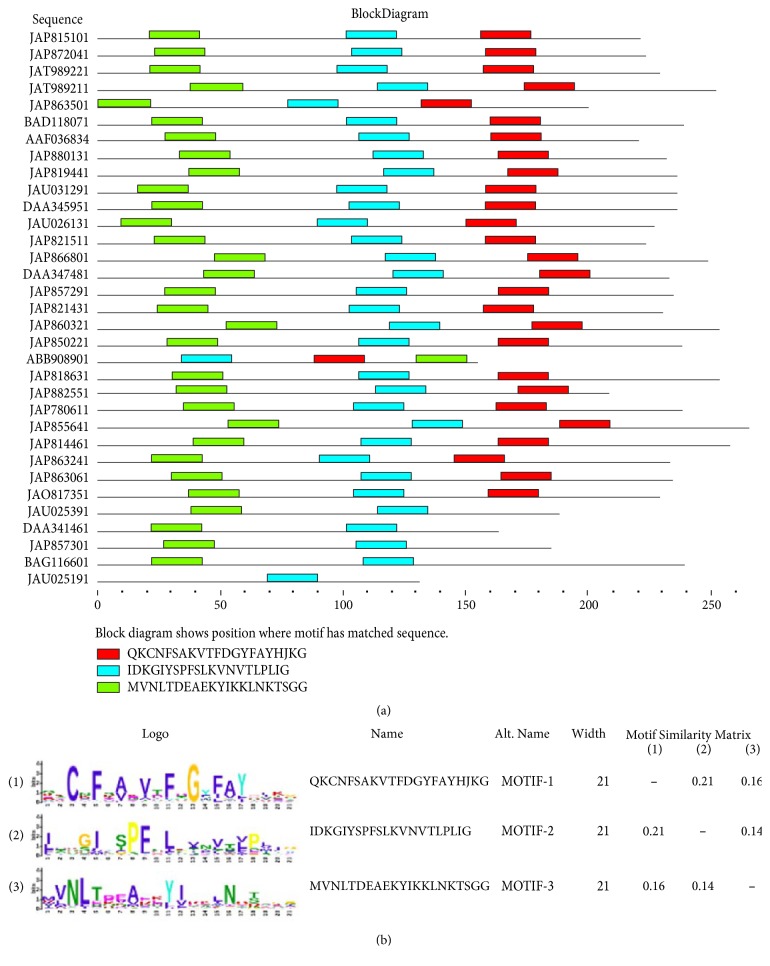
(a)* Occurrence of tandem motifs among p36 proteins*. Motif 2 is conserved across all homologs; (b)* motifs sequence logo analysis*.

The tick p36 proteins have 3 potential common motifs designated as 1, 2, and 3 with motif 2 being the only one conserved among p36 proteins (Figure 1). Motif 2 is located between amino acid positions “107–127” in the reference* D. andersoni* p36 protein. This motif 2 may be associated with a functional domain, possibly a role in immunomodulatory activity of tick p36 proteins [39]. The 3 common motifs were not found in the motif database and could be representing an orphan protein family [40].

**Figure 2:**
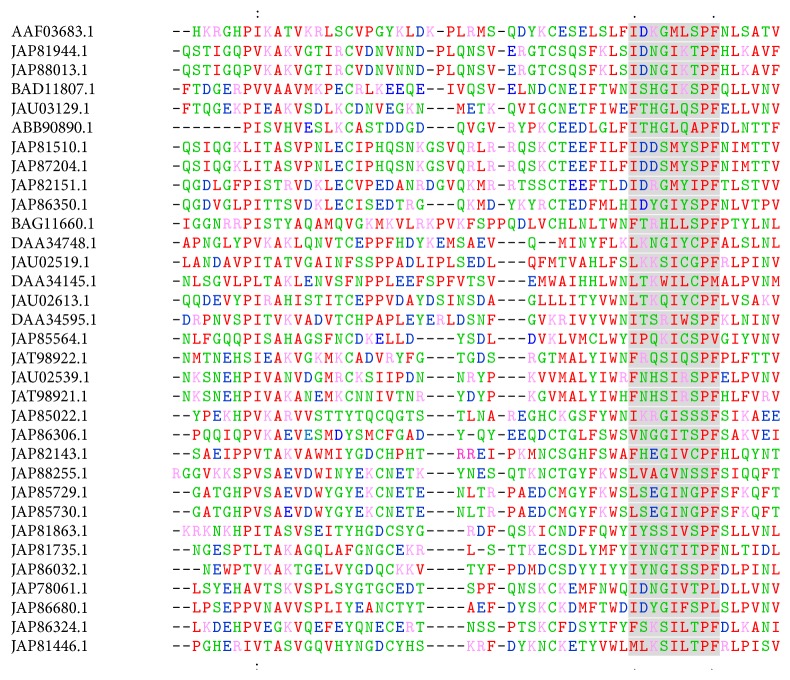
*Multiple alignment of p36 homologous amino acid sequences showing likely conservation region*. In the case of reference* D. andersoni* p36 protein, the conserved region “IDKGMLSPF” is located at positions “107–115.” (:) and (.): marks conservation between groups of strongly or weakly similar properties, respectively.* Note*. Amino acids colour according to physicochemical properties: red is for small hydrophobic, blue for acidic, magenta for basic, and green for hydroxyl/sulfhydryl/amine amino acid residues. Highlighted region shows conservation in tick p36 proteins.

**Figure 3:**
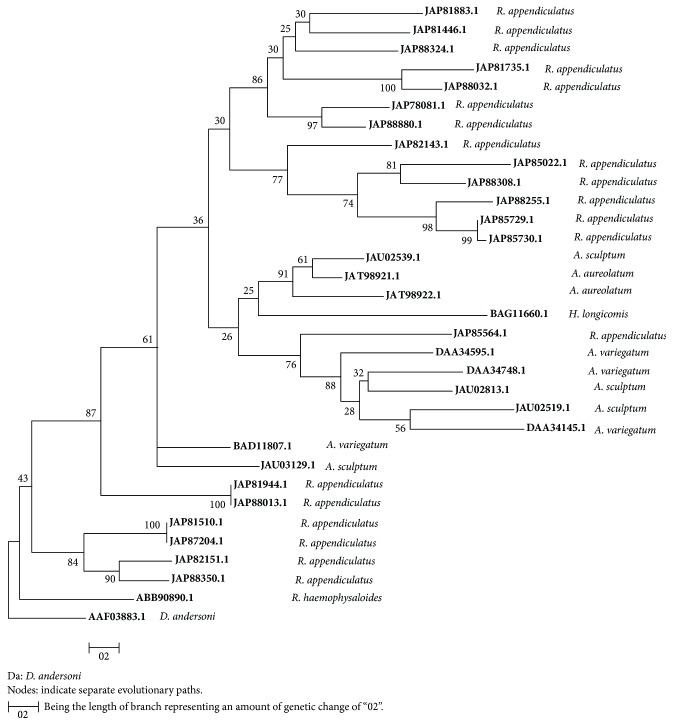
*Phylogenetic relatedness between p36 proteins*. Bootstrap resampling (1000 replicates) was employed to validate the robustness of the groupings yielded.

Alignment of tick p36 proteins revealed a conserved region occurring between amino acid positions “107–115” (“IDKGMLSPF”) in the reference* D. andersoni* p36 protein (Figure 2). This region that coincides with location of conserved motif 2 has polar amino acid residue serine (S) and charged residues lysine (K) and aspartate (D), which are associated with potential active sites [41]. Phylogenetic tree (Figure 3) showed that, among homologs with higher amino acid percentage similarity and* E*-scores,* D. andersoni* was closely related to homologs from* R. haemophysaloides* and* R. appendiculatus* as compared to homolog from* A. variegatum* indicating more recent ancestry between* Dermacentor* and* Rhipicephalus *than with* Amblyomma* genera as inferred by phylogeny [42].

### 3.2. Identification and Characterization of Antigenic Regions in the p36 Proteins

Most tick p36 proteins were predicted as secretory with signal peptide cleavage site at position 21-22 (Supplementary Table S1 and Figure S1). Secretory proteins are favoured candidates for vaccine development as they are easily accessible microbial antigens to the immune system [43].* D. andersoni* p36 protein and most p36 variants were predicted as antigenic with several homologs having antigenicity score above 0.7000 (Table 2, Supplementary Table S1), surpassing the vaxijen tool threshold of 0.5000. JAP81944.1, a homolog in* R. appendiculatus* had antigenicity score of 0.7701, comparably higher than that of Bm86 (0.7681), the constituent antigen of Tickgard and Gavac™ commercial tick vaccines. Whether this theoretically predicted immunogenicity can confer protection against tick infestation there is need to be evaluated empirically through an immunization/tick challenge set up.

The potentially conserved motif 2 in p36 protein was predicted as a likely epitope-rich antigenic region with binding residues for glycerol and lactose ligands which are associated with an immunomodulatory role [44, 45]. To facilitate tick feeding a single tick saliva protein ligand may bind receptors on several immune cell types in the vertebrate host; alternatively, multiple tick saliva proteins may bind to a common receptor [37].

### 3.3. 3D Structure of* D. andersoni* p36 Protein

*D. andersoni* p36 protein has an instability index of 35.53 and GRAVY score of −0.324 classifying it as a stable, globular protein [46]. The protein's high aliphatic index of 86.41 is associated with increase in thermostability of globular protein [47]. The stable secondary structures alpha-helix (*α*) and beta-sheets (*β*) comprised approximately 55% of* D. andersoni* p36 protein amino acid sequence (Supplementary Figure S2). The predicted conserved immunogenic region “74–107” in processed secretory* D. andersoni* p36 protein had several segments within loop regions where epitopes are generally found [48]. The combination of *α*-helixes and *β*-structures through loops with specific geometric arrangements with respect to each is responsible in forming conserved structural motifs [49, 50].

Based on Verify 3D [34] scores of the 10 models designated as 1, 2, 3, 4, 5, 6, 7, 8, 9, and 10 generated for* D. andersoni* p36 protein; models 2 and 9 were selected for further validation as they passed tool limit of 80% of the amino acids residues scoring >=0.2 in the 3D/1D profile (Supplementary Table S2). Comparison of validation scores of the selected models 2 and 9 (Table 3, Supplementary Figures S3 and S4) identified model 2 as the best 3D structure of* D. andersoni* p36 protein.

**Figure 4:**
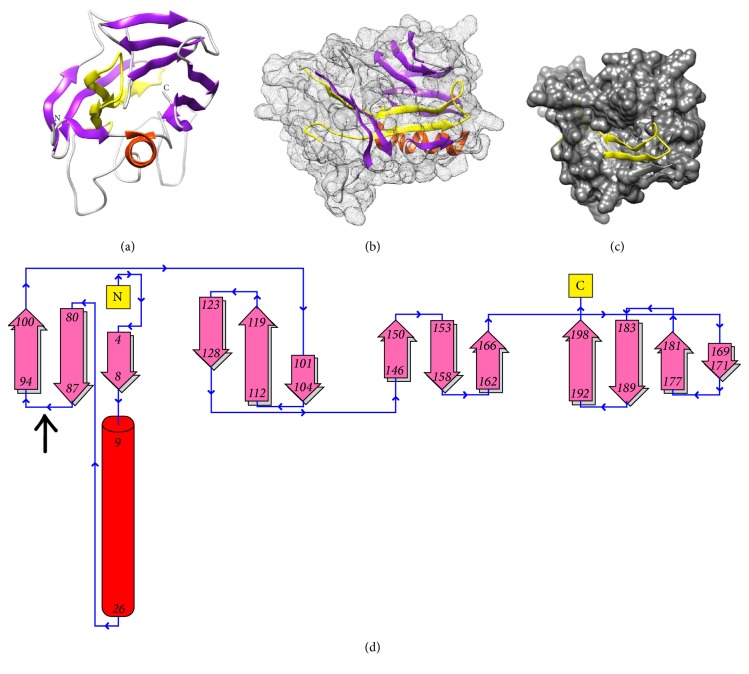
(a, b)* D. andersoni* p36 protein predicted 3D structure ribbon and space field model; (c) predicted antigenic region “74–107,” in 3D structure of* D. andersoni* p36 protein. (d) Topology of* D. andersoni* p36 protein showing the likely predicted conserved exposed loop.* Yellow*: predicted conserved antigenic region “74–107”; red: *α*-helix secondary structure; purple: *β*-strands secondary structure. ↑: predicted exposed loop region “87–94”* (“DKGMLSPF”)* in* D. andersoni* p36, showing conservation in alignment of tick p36 proteins.

The predicted 3D structure of* D*.* andersoni* p36 protein (Figures 4(a) and 4(b)) is a ball-like structure comprised of 1 alpha-helix and several antiparallel beta-strands. The region predicted as a likely conserved antigenic region “74⋯107” in* D. andersoni* p36 protein is not only located in between the alpha-helix and beta-strands but also occurs within the potentially groove region of the predicted 3D structure and further has its loop exposed on the protein surface (Figure 4(c)). Ligands bind in the largest cleft in over 83% of the proteins [51]; thus presence of the predicted conserved antigenic region within this potential groove may be associated with immunosuppressive function of* D. andersoni* p36 protein, as internal cavities in proteins are important structural elements that may produce functional motions such as ligand binding [52].

Potentially exposed loop region “87⋯94” (Figure 4(d)) in predicted 3D structure of* D. andersoni* p36 protein coincides with its likely conserved alignment region “107⋯115” after cleavage of signal peptide at amino acid position 21-22. This suggested loop region might be associated with binding site of* D. andersoni *p36 protein. The ligands predicted with potential to bind on this site include fatty acid glycerol and sugars like lactose. The hydroxyl group of polar amino acid residue serine (S), hydrophobic amino acid residue leucine (L), and charged amino acids lysine (K) and aspartic acid (D) found in this region could, respectively, have a role in binding of these ligands [53]. Immunomodulator ligands predicted with potential of binding at this site include fatty acid glycerol and sugars like lactose. There is need for future studies to evaluate whether immunomodulator ligands have a role in suppression of select T-cell receptor (TCR) induced signaling events in* D. andersoni* p36 protein mode of action [44, 45].

Collectively results from this in silico study provide further insight into potential characters of p36 protein, which is vital in exploiting the proteins as targets for developing improved next-generation cross-protective tick control approaches. In an effort to determine exact role of these proteins in tick feeding process, it is necessary for future laboratory and animal studies to confirm these preliminary predictive findings.

## 4. Conclusion

The p36 immunosuppressive proteins from ticks exhibit antigen traits worth evaluating in future experimental* in vitro* and* in vivo* trials. This includes potential conservation across several tick species and presence of a likely conserved antigenic region that may be bound by immunomodulator ligands such as glycerol and lactose. A further study is necessary on suitability of this potentially conserved region in development of a multi/chimeric antitick vaccine that incorporates critical antigenic regions. The predicted 3D model of* D. andersoni* p36 protein may be used as a template to model structures of other orphan proteins related to p36. This work is a step towards developing cross-protective next-generation antitick vaccines, as the results expand our knowledge of p36 tick saliva protein and lay ground for future studies to determine their exact role in tick feeding process, which is useful in designing blockade approaches targeting these proteins.

## Figures and Tables

**Table 1:** Tick proteins related to *D. andersoni* p36 protein.

Tick species	NCBI accession number	Sequence similarity searches						Reference
		Blastp homology search					OrthoMCL Search	
		Protein description% identityAlignmentE ValueBit score length^aa^						
*D. andersoni*	AAF03683.1	p36^*∗*^	100	220	1.00*E* − 165	459	Reference	Bergman et al., 2000
*R. appendiculatus*	JAP82151.1	Da-p36 family member	37.72	228	2.00*E* − 034	124	Co-ortholog	De castro et al., 2016
*R. appendiculatus*	JAP81510.1	Da-p36 family member	37.8	209	3.00*E* − 034	123	Co-ortholog	De castro et al., 2016
*R. appendiculatus*	JAP87204.1	Da-p36 family member	37.81	201	3.00*E* − 033	120	Co-ortholog	De castro et al., 2016
*R. appendiculatus*	JAP86350.1	Da-p36 family member	35.82	201	6.00*E* − 032	116	Co-ortholog	De castro et al., 2016
*A. variegatum*	BAD11807.1	Da-p36	35.04	234	1.00*E* − 029	111	In-paralog	Roller et al., 2004
*R. h. haemaphysaloides*	ABB90890.1	Rhh-ISP partial	36.71	158	2.00*E* − 027	103	In-paralog	Xiang et al., 2005
*A. sculptum*	JAU03129.1	Hypothetical protein partial	35.06	231	1.00*E* − 026	103	In-paralog	Eliane et al., 2016
*R. appendiculatus *	JAP81944.1	Da-p36 family member	32.3	226	2.00*E* − 024	97.4	In-paralog	De castro et al., 2016
*R. appendiculatus *	JAP88013.1	Da-p36 family member partial	31.58	228	2.00*E* − 024	97.4	In-paralog	De castro et al., 2016
*A. sculptum *	JAU02613.1	Hypothetical protein partial	27.65	217	2.00*E* − 016	74.3	In-paralog	Eliane et al., 2016
*R. appendiculatus *	JAP85022.1	Hypothetical protein	25.11	231	7.00*E* − 016	72.8	In-paralog	De castro et al., 2016
*A. sculptum *	JAU02539.1	partial Da-p36 family member	32	175	2.00*E* − 015	70.9	In-paralog	Eliane et al., 2016
*A. variegatum*	DAA34595.1	Da-p36 like	27.95	229	3.00*E* − 015	70.9	In-paralog	Ribeiro et al., 2011
*A. aureolatum*	JAT98922.1	Hypothetical protein partial	31.65	218	4.00*E* − 015	70.5	In-paralog	Martins et al., 2016
*A. aureolatum*	JAT98921.1	Hypothetical protein	30.19	212	3.00*E* − 013	65.5	In-paralog	Martins et al., 2016
*R. appendiculatus*	JAP85729.1	Da-p36 family member	26.24	221	4.00*E* − 013	65.1	In-paralog	De castro et al., 2016
*R. appendiculatus*	JAP85564.1	Da-p36 family member	25.47	212	2.00*E* − 012	63.5	In-paralog	De castro et al., 2016
*R. appendiculatus*	JAP82143.1	Da-p36 family member	23.31	236	2.00*E* − 011	60.1	In-paralog	De castro et al., 2016
*R. appendiculatus*	JAP86306.1	Da-p36 family member	25.74	237	3.00*E* − 011	60.1	In-paralog	De castro et al., 2016
*R. appendiculatus*	JAP81863.1	Da-p36 family member	27.14	210	6.00*E* − 011	59.3	In-paralog	De castro et al., 2016
*A. variegatum*	DAA34748.1	Da-p36 like	29.24	171	1.00*E* − 010	57.8	In-paralog	Ribeiro et al., 2011
*R. appendiculatus*	JAP78061.1	Da-p36 family member	26.23	244	3.00*E* − 009	54.3	In-paralog	De castro et al., 2016
*R. appendiculatus*	JAP86680.1	Da-p36 family member	27.72	202	7.00*E* − 009	53.5	In-paralog	De castro et al., 2016
*R. appendiculatus*	JAP88255.1	Da-p36 family member	26.73	202	2.00*E* − 008	52	In-paralog	De castro et al., 2016
*R. appendiculatus*	JAP85730.1	Da-p36 family member	24.82	141	2.00*E* − 008	51.2	In-paralog	De castro et al., 2016
*H. longicornis*	BAG11660.1	Isp-p36	31.78	107	1.00*E* − 007	50.1	In-paralog	Nakajima et al., 2008
*R. appendiculatus*	JAP81735.1	Da-p36 family member	24.58	240	6.00*E* − 004	38.9	In-paralog	De castro et al., 2016
*R. appendiculatus*	JAP86324.1	Da-p36 family member	24.24	198	0.001	38.1	In-paralog	De castro et al., 2016
*A. sculptum*	JAU02519.1	Hypothetical protein partial	33.7	92	0.001	36.6	In-paralog	Eliane et al., 2016
*A. variegatum*	DAA34145.1	ISP-p36 partial	28.95	76	0.002	36.6	In-paralog	Ribeiro et al., 2011
*R. appendiculatus*	JAP81446.1	Da-p36 family member	25.74	237	0.011	35	In-paralog	De castro et al., 2016
*R. appendiculatus*	JAP86032.1	Da-p36 family member	25.18	139	0.019	34.3	In-paralog	De castro et al., 2016

^aa^Amino acid; ^*∗*^reference p36 protein in *D. andersoni*.

**Table 2:** Conserved motif 2 of tick p36 proteins mapped as a potential antigenic/binding site region.

*Tick species*	*NCBI accession number*	*Antigenic score*	*Conserved Motif 2 location*	*Antigenic region* antigenic peptide tool/SVM Trip tool	*Mapped immunogenic segments of Motif 2* Epitopia tool	*Mapped Binding sites of Motif 2* Sprint pep tool	*Predicted ligands for Motif 2* Coach tool
*D. andersoni*	AAF03683.1	0.5880	107–127	95–128	I**DKG**MLSPFNLSA**T**V**K**FPLI**P**	I**DKG**M**L**SP**F**NL**S**A**T**VKF**P**LIP	Lactose, Glycerol, NAG-(4-1)GAL, Sucrose

*R. appendiculatus*	JAP81944.1	0.7701	117–137	108–137	I**DNG**I**K**TPF**H**LKAVFSFPIT**G**	**IDNG**I**K**TP**F**HL**K**AVF**S**F**P**ITG	-

*R. appendiculatus*	JAP88013.1	0.7379	113–133	104–133	I**DNG**I**K**TPF**H**LKAVFSFPIT**G**	**IDNG**I**K**TP**F**HL**K**AVF**S**F**P**ITG	-

*R. appendiculatus*	JAP81510.1	0.7258	102–122	94–133	IDDSMYSPFNIMTTVAFPLI**G**	**IDDSMY**S**PF**N**IM**T**T**VAF**P**LIG	B-Octylglucoside

*R. appendiculatus*	JAP86350.1	0.7072	78–98	74–110	IDY**G**IYSPFNLVTPVQFPLMG	I**DYG**I**Y**S**PF**N**LV**TPV**Q**F**P**LMG	Alpha-D-Mannose, Alpha-D-Lactose, B-Octylglucoside

Bold sections: tick p36 conserved region residues mapped as potentially antigenic/binding sites.

**Table 3:** Validation of 3D structures for models 2 and 9 of *D. andersoni* p36 protein.

Number	Validation tool	Parameter monitored	Limit	Results	
				Model 2	Model 9
(1)	*ProQPsiPred*	LGscore	LG score > 1.5 fairly good model LG score > 2.5 very good model LG score > 4 extremely good model	LGscore 2.797	LGscore 2.366
		MaxSub	MaxSub > 0.1 fairly good model MaxSub > 0.5 very good model MaxSub > 0.8 extremely good model	MaxSub 0.208	MaxSub 0.055

(2)	*ProQJPred*	LGscore	LG score > 1.5 fairly good model LG score > 2.5 very good model LG score > 4 extremely good model	LGscore 2.914	LGscore 2.231
		MaxSub	MaxSub > 0.1 fairly good model MaxSub > 0.5 very good model MaxSub > 0.8 extremely good model	MaxSub 0.219	MaxSub 0.046

(3)	*Rampage tool*	Favoured region residues Allowed region residues Outlier region residues		156 (79.2%) 26 (13.2%) 15 (7.6%)	152 (77.2%) 24 (12.2%) 21 (10.7%)

## Supplementary Materials

Supplementary Materials Supplementary Table S1: protein targeting pathway and antigenic potential of tick p36 proteins. Supplementary Table S2: Verify 3D validation scores of models generated for* D. andersoni* p36 protein. Supplementary Figure S1:* D. andersoni* p36 protein signal peptide cleavage site location. Supplementary Figure S2: Spider^2^ tool secondary structure characterization of* D. andersoni* p36 protein. Supplementary Figure S3: rampage tool assessment of Ramachandran plot for model 2 of* D. andersoni* p36 protein. Supplementary Figure S4: rampage tool assessment of Ramachandran plot for model 9 of* D. andersoni* p36 protein.

## References

[1] De La Fuente J., Estrada-Pena A., Venzal J. M., Kocan K. M., Sonenshine D. E. (2008). Overview: Ticks as vectors of pathogens that cause disease in humans and animals. *Frontiers in Bioscience*.

[2] Jongejan F., Uilenberg G. (2004). The global importance of ticks. *Parasitology*.

[3] Abbas R. Z., Zaman M. A., Colwell D. D., Gilleard J., Iqbal Z. (2014). Acaricide resistance in cattle ticks and approaches to its management: The state of play. *Veterinary Parasitology*.

[4] Fuente J., Kocan K. M., Blouin E. F. (2007). Tick vaccines and the transmission of tick-borne pathogens. *Veterinary Research Communications*.

[5] Merino O., Alberdi P., Pérez De La Lastra J. M., de la Fuente J. (2013). Tick vaccines and the control of tick-borne pathogens. *Frontiers in Cellular and Infection Microbiology*.

[6] De La Fuente J., Contreras M. (2015). Tick vaccines: Current status and future directions. *Expert Review of Vaccines*.

[7] Oldiges D. P., Laughery J. M., Tagliari N. J. (2016). Transfected Babesia bovis Expressing a Tick GST as a Live Vector Vaccine. *PLOS Neglected Tropical Diseases*.

[8] García-García J. C., Montero C., Redondo M. (2000). Control of ticks resistant to immunization with Bm86 in cattle vaccinated with the recombinant antigen Bm95 isolated from the cattle tick, Boophilus microplus. *Vaccine*.

[9] Odongo D., Kamau L., Skilton R. (2007). Vaccination of cattle with TickGARD induces cross-reactive antibodies binding to conserved linear peptides of Bm86 homologues in Boophilus decoloratus. *Vaccine*.

[10] Nuttall P. A., Trimnell A. R., Kazimirova M., Labuda M. (2006). Exposed and concealed antigens as vaccine targets for controlling ticks and tick-borne diseases. *Parasite Immunology*.

[11] Fontaine A., Pascual A., Diouf I. (2011). Mosquito salivary gland protein preservation in the field for immunological and biochemical analysis. *Parasites & Vectors*.

[12] Leboulle G., Crippa M., Decrem Y. (2002). Characterization of a novel salivary immunosuppressive protein from Ixodes ricinus ticks. *The Journal of Biological Chemistry*.

[13] Titus R. G., Bishop J. V., Mejia J. S. (2006). The immunomodulatory factors of arthropod saliva and the potential for these factors to serve as vaccine targets to prevent pathogen transmission. *Parasite Immunology*.

[14] Anguita J., Ramamoorthi N., Hovius J. W. R. (2002). Salp15, an Ixodes scapularis salivary protein, inhibits CD4+ T cell activation. *Immunity*.

[15] Dai J., Wang P., Adusumilli S. (2009). Antibodies against a tick protein, Salp15, protect mice from the Lyme disease agent. *Cell Host & Microbe*.

[16] Almazán C., Moreno-Cantú O., Moreno-Cid J. A. (2012). Control of tick infestations in cattle vaccinated with bacterial membranes containing surface-exposed tick protective antigens. *Vaccine*.

[17] Moreno-Cid J. A., Pérez de la Lastra J. M., Villar M. (2013). Control of multiple arthropod vector infestations with subolesin/akirin vaccines. *Vaccine*.

[18] Parizi L. F., Githaka N. W., Logullo C. (2012). The quest for a universal vaccine against ticks: Cross-immunity insights. *The Veterinary Journal*.

[19] Alarcon-Chaidez F. J., Müller-Doblies U. U., Wikel S. (2003). Characterization of a recombinant immunomodulatory protein from the salivary glands of Dermacentor andersoni. *Parasite Immunology*.

[20] Bergman D. K., Palmer M. J., Caimano M. J., Radolf J. D., Wikel S. K. (2000). Isolation and molecular cloning of a secreted immunosuppressant protein from Dermacentor andersoni salivary gland. *Journal of Parasitology*.

[21] Nene V., Lee D., Quackenbush J. (2002). AvGI, an index of genes transcribed in the salivary glands of the ixodid tick Amblyomma variegatum. *International Journal for Parasitology*.

[22] Nene V., Lee D., Kang'A S. (2004). Genes transcribed in the salivary glands of female Rhipicephalus appendiculatus ticks infected with Theileria parva. *Insect Biochemistry and Molecular Biology*.

[23] Konnai S., Nakajima C., Imamura S. (2009). Suppression of cell proliferation and cytokine expression by HL-p36, a tick salivary gland-derived protein of Haemaphysalis longicornis. *The Journal of Immunology*.

[24] Wang F., Lu X., Guo F. (2017). The immunomodulatory protein RH36 is relating to blood-feeding success and oviposition in hard ticks. *Veterinary Parasitology*.

[25] Parizi L. F., Reck J., Oldiges D. P. (2012). Multi-antigenic vaccine against the cattle tick Rhipicephalus (Boophilus) microplus: A field evaluation. *Vaccine*.

[26] Kulp D. W., Schief W. R. (2013). Advances in structure-based vaccine design. *Current Opinion in Virology*.

[27] Altschul S. F., Gish W., Miller W., Myers E. W., Lipman D. J. (1990). Basic local alignment search tool. *Journal of Molecular Biology*.

[28] Fischer S., Brunk B. P., Chen F. (2011). Using OrthoMCL to assign proteins to OrthoMCL-DB groups or to cluster proteomes into new ortholog groups. *Current Protocols in Bioinformatics*.

[29] Li L., Stoeckert C. J., Roos D. S. (2003). OrthoMCL: identification of ortholog groups for eukaryotic genomes. *Genome Research*.

[30] Whelan S., Goldman N. (2001). A general empirical model of protein evolution derived from multiple protein families using a maximum-likelihood approach. *Molecular Biology and Evolution*.

[31] Zuckerkandl E., Pauling L. (1965). *Evolutionary divergence and convergence in proteins*.

[32] Yao B., Zhang L., Liang S., Zhang C. (2012). SVMTriP: a method to predict antigenic epitopes using support vector machine to integrate tri-peptide similarity and propensity. *PLoS ONE*.

[33] Xu D., Zhang Y. (2012). Ab initio protein structure assembly using continuous structure fragments and optimized knowledge-based force field. *Proteins: Structure, Function, and Bioinformatics*.

[34] Eisenberg D., Lüthy R., Bowie J. U. (1997). VERIFY3D: assessment of protein models with three-dimensional profiles. *Methods in Enzymology*.

[35] Xu D., Zhang Y. (2011). Improving the physical realism and structural accuracy of protein models by a two-step atomic-level energy minimization. *Biophysical Journal*.

[36] de la Fuente J., Almazán C., Blas-Machado U. (2006). The tick protective antigen, 4D8, is a conserved protein involved in modulation of tick blood ingestion and reproduction. *Vaccine*.

[37] Chmelař J., Kotál J., Kopecký J., Pedra J. H. F., Kotsyfakis M. (2016). All For One and One For All on the Tick-Host Battlefield. *Trends in Parasitology*.

[38] Rangel C. K., Parizi L. F., Sabadin G. A. (2017). Molecular and structural characterization of novel cystatins from the taiga tick Ixodes persulcatus. *Ticks and Tick-borne Diseases*.

[39] Sleator R. D., Walsh P. (2010). An overview of in silico protein function prediction. *Archives of Microbiology*.

[40] Tautz D., Domazet-Lošo T. (2011). The evolutionary origin of orphan genes. *Nature Reviews Genetics*.

[41] Reddy B. V. B., Li W. W., Shindyalov I. N., Bourne P. E. (2001). Conserved key amino acid positions (CKAAPs) derived from the analysis of common substructures in proteins. *Proteins: Structure, Function and Genetics*.

[42] Hoogstraal H., Aeschlimann A. (1982). Tick-Host Specificity. Bull. La Société Entomol. *Suisse*.

[43] Bendtsen J. D., Wooldridge K. G. (2009). *Bacterial secreted proteins: Secretory mechanisms and role in pathogenesis*.

[44] Zhang M. S., Sandouk A., Houtman J. C. D. (2016). Glycerol Monolaurate (GML) inhibits human T cell signaling and function by disrupting lipid dynamics. *Scientific Reports*.

[45] Paasela M., Kolho K.-L., Vaarala O., Honkanen J. (2014). Lactose inhibits regulatory T-cell-mediated suppression of effector T-cell interferon-*γ* and IL-17 production. *British Journal of Nutrition*.

[46] Gasteiger E., Hoogland C., Gattiker A. (2005). Protein Identification and Analysis Tools on the ExPASy Server. *The Proteomics Protocols Handbook*.

[47] Ikai A. (1980). Thermostability and Aliphatic Index of Globular Proteins. *The Journal of Biochemistry*.

[48] Dalkas G. A., Teheux F., Kwasigroch J. M., Rooman M. (2014). Cation-*π*, amino-*π*, *π*-*π*, and H-bond interactions stabilize antigen-antibody interfaces. *Proteins: Structure, Function, and Bioinformatics*.

[49] Cowen L., Bradley P., Menke M., King J., Berger B. (2002). Predicting the beta-helix fold from protein sequence data. *Journal of Computational Biology*.

[50] Conte M. R., Grüne T., Ghuman J. (2000). Structure of tandem RNA recognition motifs from polypyrimidine tract binding protein reveals novel features of the RRM fold. *EMBO Journal*.

[51] Laskowski R. A., Luscombe N. M., Swindells M. B., Thornton J. M. (1996). Protein clefts in molecular recognition and function. *Protein Science*.

[52] Ogata K., Kanei-Ishii C., Sasaki M. (1996). The cavity in the hydrophobic core of Myb DNA-binding domain is reserved for DNA recognition and trans-activation. *Nature Structural & Molecular Biology*.

[53] Barnes M. R., Gray I. C. (2003). *Bioinformatics for Geneticists*.

