# Supplementary material for: In Silico Characterization and Structural Modeling of Dermacentor andersoni p36 Immunosuppressive Protein
Source: Adv Bioinformatics. 2018 Apr 8;2018:7963401. doi: 10.1155/2018/7963401 (PMC5911333; doi:10.1155/2018/7963401)

**Supplementary Table S1:** Protein targeting pathway and antigenic potential of tick p36 proteins

| **Tick species** | **NCBI**  **Accession**  **No.** | **Protein Description** | **Protein targeting** | | | | **Antigenic potential**  Model: Parasite  Threshold: 0.5 | |
| --- | --- | --- | --- | --- | --- | --- | --- | --- |
|  |  |  | **Secretory proteins**  **(Signalp tool)** | | **Surface proteins** | | **Antigenic score** | **Remarks** |
|  |  |  | Signal  peptide  presence | Cleavage site | TM Protein (TMHMM tool) | GPI anchored Protein  (PredGPI tool) |  |  |
| *D. andersoni* | AAF03683.1 | p36* | Yes | 21-22 | 0 | No | 0.5880 | Probable antigen |
| *R. appendiculatus* | JAP82151.1 | Da-p36 family member | Yes | 21-22 | 0 | No | 0.5468 | Probable antigen |
| *R. appendiculatus√* | JAP81510.1 | Da-p36 family member | Yes | 20-21 | 0 | No | 0.7258 | Probable antigen |
| *R. appendiculatus* | JAP87204.1 | Da-p36 family member | No |  | 0 | No | 0.6137 | Probable antigen |
| *R. appendiculatus√* | JAP86350.1 | Da-p36 family member | No |  | 0 | No | 0.7072 | Probable antigen |
| *A. variegatum* | BAD11807.1 | Da-p36 | Yes | 21-22 | 0 | No | 0.5644 | Probable antigen |
| *R. h. haemaphysaloides* | ABB90890.1 | Rhh-ISP partial | No |  | 0 | No | 0.4523 | Probable non-antigen |
| *A. sculptum* | JAU03129.1 | Hypothetical protein partial | Yes | 13-14 | 0 | No | 0.3102 | Probable non-antigen |
| *R. appendiculatus√* | JAP81944.1 | Da-p36 family member | No |  | 1 | No | 0.7701 | Probable antigen |
| *R. appendiculatus√* | JAP88013.1 | Da-p36 family member partial | No |  | 1 | No | 0.7379 | Probable antigen |
| *A. sculptum* | JAU02613.1 | Hypothetical protein partial | No |  | 0 | No | 0.3865 | Probable non-antigen |
| *R. appendiculatus* | JAP85022.1 | Da-p36 family member | Yes | 17-18 | 0 | No | 0.5687 | Probable antigen |
| *A. sculptum* | JAU02539.1 | Hypothetical protein partial | Yes | 18-19 | 0 | No | 0.5381 | Probable antigen |
| *A. variegatum* | DAA34595.1 | Da-p36 like | Yes | 21-22 | 0 | No | 0.5012 | Probable antigen |
| *A. aureolatum* | JAT98922.1 | Hypothetical protein partial | No |  | 0 | No | 0.5026 | Probable antigen |
| *A. aureolatum* | JAT98921.1 | Hypothetical protein | Yes | 18-19 | 0 | No | 0.4439 | Probable non-antigen |

**Table S1 cont…..**

| *R. appendiculatus* | | JAP85729.1 | Da-p36 family member | | No |  | 0 | No | 0.5000 | Probable antigen | |
| --- | --- | --- | --- | --- | --- | --- | --- | --- | --- | --- | --- |
| *R. appendiculatus* | | JAP85564.1 | Da-p36 family member | | Yes | 22-23 | 0 | No | 0.5569 | Probable antigen | |
| *R. appendiculatus* | | JAP82143.1 | Da-p36 family member | | No |  | 0 | No | 0.3930 | Probable non-antigen | |
| *R. appendiculatus* | | JAP86306.1 | Da-p36 family member | | Yes | 24-25 | 0 | No | 0.5162 | Probable antigen | |
| *R. appendiculatus* | | JAP81863.1 | Da-p36 family member | | No |  | 0 | No | 0.5856 | Probable antigen | |
| *A. variegatum* | | DAA34748.1 | Da-p36 like | | Yes | 21-22 | 0 | No | 0.5733 | Probable antigen | |
| *R. appendiculatus* | | JAP78061.1 | Da-p36 family member | | Yes | 21-22 | 0 | No | 0.3387 | Probable non-antigen | |
| *R. appendiculatus* | | JAP86680.1 | Da-p36 family member | | Yes | 21-22 | 0 | No | 0.5962 | Probable antigen | |
| *R. appendiculatus* | | JAP88255.1 | Da-p36 family member | | No |  | 1 | No | 0.3636 | Probable non-antigen | |
| *R. appendiculatus* | | JAP85730.1 | Da-p36 family member | | No |  | 0 | No | 0.3551 | Probable non-antigen | |
| *H. longicornis* | | BAG11660.1 | Isp-p36 | | Yes | 21-22 | 0 | No | 0.6248 | Probable antigen | |
| *R. appendiculatus* | | JAP81735.1 | Da-p36 family member | | Yes | 21-22 | 0 | No | 0.5229 | Probable antigen | |
| *R. appendiculatus* | | JAP86324.1 | Da-p36 family member | | Yes | 19-20 | 0 | No | 0.5781 | Probable antigen | |
| *A. sculptum* | JAU02519.1 | | | Hypothetical protein partial | No |  | 0 | No | 0.5704 | Probable antigen |  |
| *A. variegatum* | DAA34145.1 | | | ISP-p36 partial | Yes | 21-22 | 0 | No | 0.5138 | Probable antigen |  |
| *R. appendiculatus* | JAP81446.1 | | | Da-p36 family member | Yes | 21-22 | 0 | No | 0.3690 | Probable non-antigen |  |
| *R. appendiculatus* | JAP86032.1 | | | Da-p36 family member | Yes | 21-22 | 0 | No | 0.4766 | Probable non-antigen |  |
| *R. microplus√* | ADQ19690.1 | | | *Bm86 glycoprotein |  |  |  |  | 0.7681 | Probable antigen |  |

Key:

√- Proteins with antigenicty score above 0.7000

*Bm 86 glycoprotein a known anti-tick vaccine antigen from *R. microplus* used as reference.

- TM (Transmembrane)

- TMHMM (Transmembrane hidden Markov model)

- GPI (Glycosylphosphatidylinositol)

**Supplementary Table S2:** Verify 3D validation scores of models generated for *D. andersoni* p36 protein

| **3-D structure models** | **Verify 3D Score** | **Remarks** |
| --- | --- | --- |
| Model 1 | 49.75% of the residues had an averaged 3D-1D score >= 0.2 | Error |
| Model 2 | 81.41% of the residues had an averaged 3D-1D score >= 0.2 | Pass |
| Model 3 | 69.35% of the residues had an averaged 3D-1D score >= 0.2 | Error |
| Model 4 | 61.81% of the residues had an averaged 3D-1D score >= 0.2 | Error |
| Model 5 | 57.29% of the residues had an averaged 3D-1D score >= 0.2 | Error |
| Model 6 | 68.34% of the residues had an averaged 3D-1D score >= 0.2 | Error |
| Model 7 | 53.27% of the residues had an averaged 3D-1D score >= 0.2 | Error |
| Model 8 | 61.31% of the residues had an averaged 3D-1D score >= 0.2 | Error |
| Model 9 | 88.44% of the residues had an averaged 3D-1D score >= 0.2 | Pass |
| Model 10 | 60.80% of the residues had an averaged 3D-1D score >= 0.2 | Error |

Key:

Parameter Monitored: Determines compatibility of an atomic model (3D) with its own amino acid sequence (1D)

Limit: Pass, At least 80% of the amino acids have scored >= 0.2 in the 3D/1D profile

**Supplementary Figure S1:** *D. andersoni* p36 protein signal peptide cleavage site location


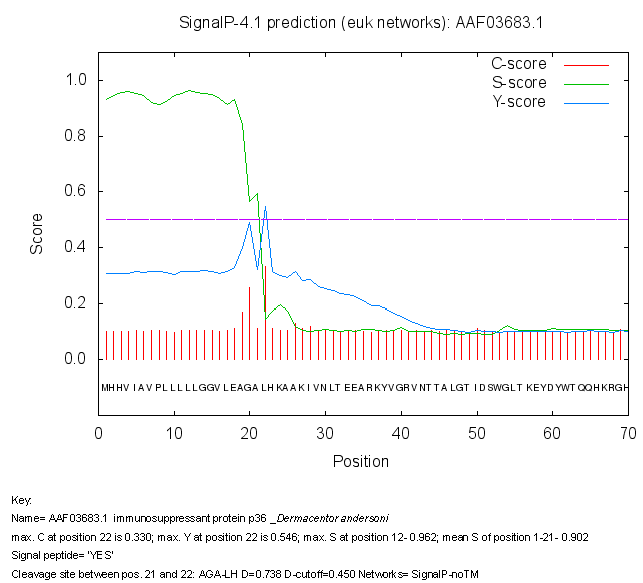


**Supplementary Figure S2:** Spider^2^ tool secondary structure characterization of *D. andersoni* p36 protein


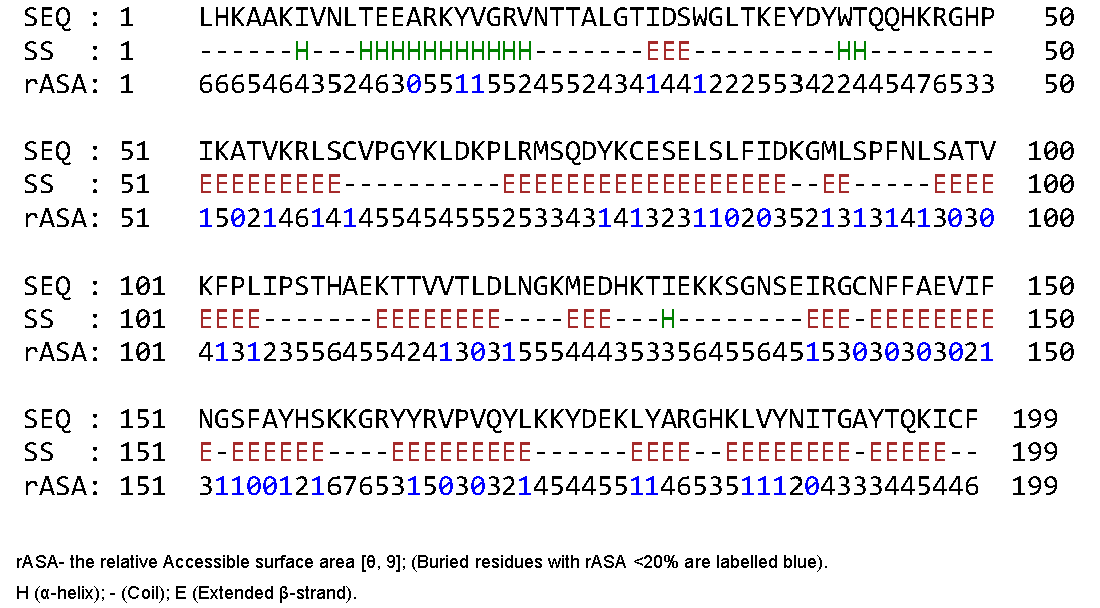


Among 199 residues of *D. andersoni* p36 protein sequence, 15 are predicted as α-helix and 95 as β-strands. Predicted immunogenic region “74…107” (“QDYKCESELSLFIDKGMLSPFNLSATVKFPLIPS”), has its segments “KG”, “SPFNL” and “IPS” in loop regions.

**Supplementary Fig S3:** Rampage tool assessment of Ramachandran plot for model 2 of *D. andersoni* p36 protein


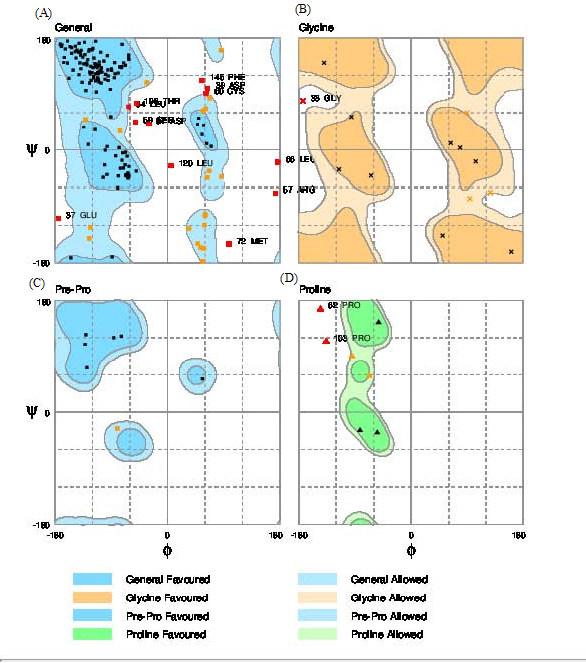


Ramachandran plot showing the distribution of backbone configuration of residue in model 2. (A) All amino acids other than proline and glycine; (B) Glycine residues; (C) Residues preceeding proline in the amino acid sequence; (D) Proline residues.


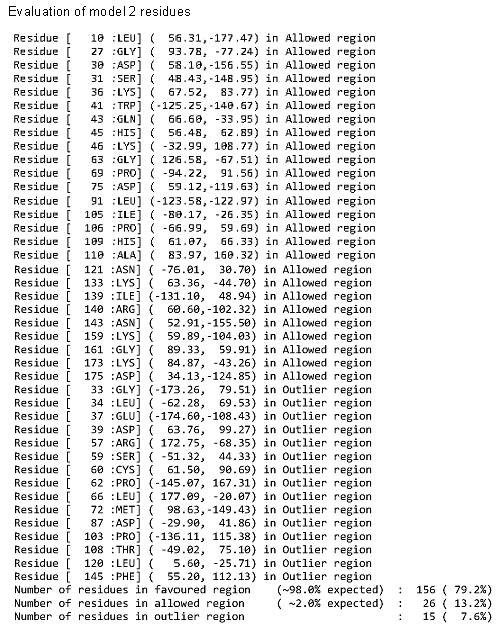


**Supplementary Fig S4:** Rampage tool assessment of Ramachandran plot for model 9 of *D. andersoni* p36 protein


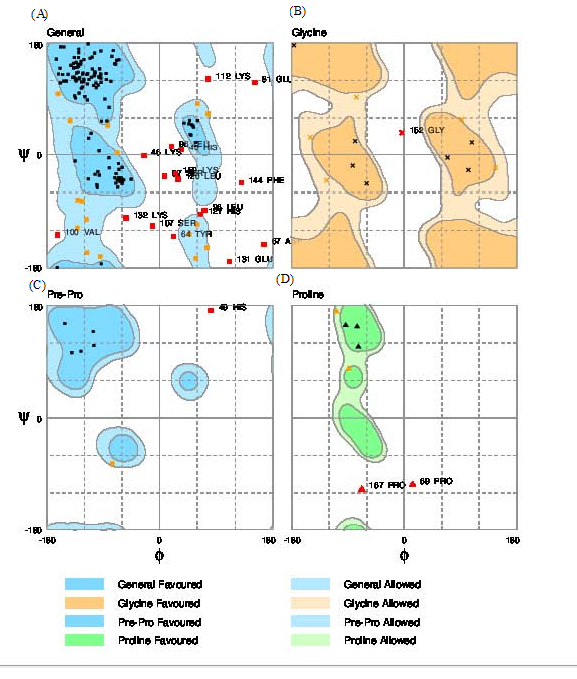


Ramachandran plot showing the distribution of backbone configurations of residue in model 9. (A) All amino acids other than proline and glycine; (B)Glycine residues; (C) Residues preceeding proline in the amino acid sequence; (D) Proline residues.


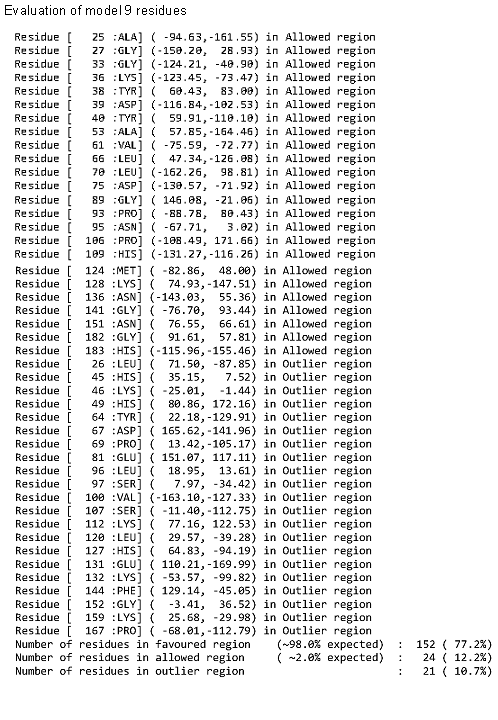

Supplement: Supplementary Materials — Supplementary Table S1: protein targeting pathway and antigenic potential of tick p36 proteins. Supplementary Table S2: Verify 3D validation scores of models generated for D. andersoni p36 protein. Supplementary Figure S1: D. andersoni p36 protein signal peptide cleavage site location. Supplementary Figure S2: Spider2 tool secondary structure characterization of D. andersoni p36 protein. Supplementary Figure S3: rampage tool assessment of Ramachandran plot for model 2 of D. andersoni p36 protein. Supplementary Figure S4: rampage tool assessment of Ramachandran plot for model 9 of D. andersoni p36 protein. [file 7963401.f1.docx]
